# Current Perspectives of the Chicken Gastrointestinal Tract and Its Microbiome

**DOI:** 10.1016/j.csbj.2018.03.002

**Published:** 2018-03-15

**Authors:** Daniel Borda-Molina, Jana Seifert, Amélia Camarinha-Silva

**Affiliations:** Institute of Animal Science, University of Hohenheim, 70599 Stuttgart, Germany

**Keywords:** Chicken broilers, Microbiome, Gastrointestinal tract, Omics, Microbiome-host interaction

## Abstract

The microbial communities inhabiting the gastrointestinal tract (GIT) of chickens are essential for the gut homeostasis, the host metabolism and affect the animals' physiology and health. They play an important role in nutrient digestion, pathogen inhibition and interact with the gut-associated immune system.

Throughout the last years high-throughput sequencing technologies have been used to analyze the bacterial communities that colonize the different sections of chickens' gut. The most common methodologies are targeted amplicon sequencing followed by metagenome shotgun sequencing as well as metaproteomics aiming at a broad range of topics such as dietary effects, animal diseases, bird performance and host genetics. However, the respective analyses are still at the beginning and currently there is a lack of information in regard to the activity and functional characterization of the gut microbial communities. In the future, the use of multi-omics approaches may enhance research related to chicken production, animal and also public health. Furthermore, combinations with other disciplines such as genomics, immunology and physiology may have the potential to elucidate the definition of a “healthy” gut microbiota.

## 1 Introduction

The global population is increasing continuously and is estimated to comprise about 9.6 billion individuals by 2050. Correspondingly, poultry production has intensified during the last years and is predicted to produce about 130 million tons of chicken meat in 2020 (OECD/FAO) to match the demands of a growing world population. Such extreme growth is only feasible with proper strategies for disease control and prevention to minimize the impact of bacterial, parasitic or viral infections of the animals and simultaneously reduce associated ecological damage and waste of resources.

Chicken breeders focused on high performance, fast growth, breast meat yield, efficiency of feed conversion rates, skeletal quality, heart and lung functionality and as well on egg production and quality. Looking for the preferred phenotypic traits and selecting the most superior individuals influenced the animals' genetics [1]. However, selection for a single trait may also affect other traits. For example, broiler chickens that were selected for meat production gained a higher body weight (~3 kg) within 42 days. On the other hand, ascites and/or lameness occurred in the animals [2]. Thus, a balanced selection across the different traits might improve the animals' well-being.

Besides breeding and selection, optimized nutrition of broiler chickens is a fundamental component of efficient poultry production. The animals' fodder accounts for 70% of the total costs in chicken production [3] and poultry diets are expensive since egg and meat production require high amounts of energy and protein sources. Diets contain energy and protein, mineral supplements, specific amino acids and vitamins in a defined formulation providing all nutrients necessary for the bird's health and adequate performance. Diets with imbalanced mineral supplementation may lead to health problems and result in inefficient use of the natural resources. Consequently, high amounts of valuable nutrients such as nitrogen, phosphorus (P), calcium (Ca) and zinc get lost by defecation and urination [4].

Gut microorganisms are mainly responsible for the degradation of complex substrates such as non-starch polysaccharides which requires highly specialized, hydrolytic enzymes [5]. The discovery of novel enzymatic tools depends on metagenomic data for instance from the broiler caeca. Recently, a xylanase gene from the chicken caecum has been isolated and overexpressed which emphasizes the potential for the development of new, optimized feed additives for industrial application [6]. Close interactions between the intestinal microbiome and the animals' diet are well established since dietary factors are known to alter the gut microbiota. Bacteria are able to hydrolyze indigestible carbohydrates and polysaccharides allowing further fermentation by other members of the gut ecosystem that produce short chain fatty acids (SCFA) which in turn become available for the host.

Moreover, microorganisms growing on poultry litter have an influence on the gut microbiome and may constitute a source of infection. Since the first day of life, chicks start pecking and ingesting litter materials including the adhered microorganisms that are usually detected in feces and soil. In this way, microbes of other habitats can be transferred to the gastrointestinal tract [7]. Previous studies have shown that *Salmonella* and *Clostridium perfringens* decrease in abundance in reused litter and *Campylobacter jejuni* and *Escherichia coli* become more prevalent [7]. Wang et al. compared the microbiota of fresh and reused litter and its effects on the chickens' gut microbiota finding an increase of halotolerant/alkaliphilic bacteria in reused litter and a stronger effect of the litter on the microbiota of the ileum in comparison to the caecal microbiota. Caecal samples of young birds raised in reused litter showed a higher bacterial diversity when compared to mature animals that were kept under the same conditions. The reuse of litter is a common practice in broiler production. Despite studies showing that reused litter does not exhibit higher abundances of *C. perfringens* or *Salmonella* [8], chickens raised in fresh litter revealed an increasing colonization with beneficial *Lactobacillus* spp. [9]. Proper litter management may reduce pathogen activity, promote a balanced gut microbiome and improve the chickens' health status.

This review will focus on the methodologies that were used in the past years to characterize the microbial communities within the chickens' gut to provide insights into the effects of different feeding strategies and host genetics on the gut microbiome. New perspectives will elucidate yet unknown aspects of the chickens' gut microbiome.

## 2 Exploring the Composition and Function of the Chicken Gut Microbiome

### 2.1 Targeted Amplicon Sequencing of the 16S rRNA Gene

Next-generation sequencing revolutionized the characterization of microbial communities. The respective studies are mainly based on amplifying the small subunits of the 16S ribosomal gene of Bacteria and Archaea, the 18S rRNA gene of eukaryotic species and the nuclear ribosomal internal transcribed spacer (ITS) regions of Fungi [10]. In this way, deep characterization of microbial communities and quantification of relative abundances of the different microorganisms can be achieved. Most of the studies available aim at the bacterial 16S rRNA gene. Even though this method has been used in other scientific disciplines for several years, the first study characterizing the chickens' gastrointestinal microbiota was published in 2011 [11]. The 16S rRNA gene comprises nine hypervariable regions [12]. However, so far microbial studies of the chickens' gut have covered the V1–V3, V3–V4, V4–V5, V1, V3 or V4 regions [5,7,11,[13], [14], [15], [16], [17], [18]]. The sequencing technologies of choice are Roche 454-pyrosequencing, Illumina MiSeq, HiSeq and Ion PGM systems [19]. Bioinformatic processing of the generated sequences can be achieved by employing open sources platforms such as QIIME [20] and mothur [21] that, in order to perform taxonomic assignments, depend on public databases like GreenGenes [22], the ribosomal database project (RDP) [23] and SILVA [24]. The latter represents the most recent database. Functional prediction algorithms such as PICRUSt and Tax4Fun can be used to obtain further information from 16S rRNA gene sequencing data. PICRUSt is based on the GreenGenes database and uses an algorithm with proved accuracy regarding humans, soils and mammalian guts [25]. However, the GreenGenes database was last updated in 2013. Tax4Fun employs the SILVA database and claims to reach higher correlations regarding the functional predictions since the link association is based on the nearest neighbor with a minimum sequence similarity. Despite the promising information that can be obtained by functional prediction processing, caution is advised when drawing strong conclusions since there are large numbers of operational taxonomic units (OTUs) that cannot be assigned to a specific genus and not even to a family level [31]. Moreover, the respective approaches should be validated thoroughly in particular for avian species since their deviating organism may imply different functions and associations between microorganisms and the host.

More than 900 bacterial species inhabit the GIT of broilers being involved in the digestion of food, breakdown of toxins, stimulation of the immune system, exclusion of pathogens and endocrine activity. Interactions between microorganisms and the GIT influence the stability of the microbial communities, the animals' health, growth and consequently also feed conversion rates [26]. As feed is ingested and moves through the GIT, different groups of microbes start the digestion. The chickens' GIT is divided into three parts: the upper segment, small intestine and large intestine that are colonized by microbes in their entire length. Due to the enormous diversification of each GIT section, they are commonly studied as independent ecosystems. However, it is known that the different sections are highly interconnected and thus also influence each other's community composition [27]. Variations regarding the protocols for DNA extraction, choice of the amplified 16S rRNA gene regions and overall microbial community characterization make comparison between studies difficult. The study design strongly influences the microbial profiles of each gut section due to the differences between individual birds, species, gender, age, genetics, diets and housing. Microbiota studies in individual chickens showed a high inter-individual variation, disregarding the identical diet composition or housing conditions [5,13,16].

In the crop, breakdown of starch and lactate fermentation are initiated by several *Lactobacillus* sp. and *Bifidobacterium* sp. as well as by members of the Enterobacteriaceae family that were also detected within this section [28]. Lactobacilli also appear in high abundances in the proventriculus and gizzard. Nutrient absorption occurs in the ileum which exhibits high numbers of *Lactobacillus* sp. and to a lesser extend bacteria with butyrate producing activities such has *Clostridium*, *Streptococcus* and *Enterococcus* [28]. Fermentation and digestion of complex substrates such as cellulose, starch and other polysaccharides occur in the caecum, which is the most diverse gut section characterized by the longest feed retention time (12–20 h). In contrast, only 2.5 h are required to pass through the upper parts of the intestine [36]. The most abundant families within the caecum are Clostridiaceae, Bacteroidaceae, Lactobacillaceae and butyrate producers like Lachnospiraceae. The caecum is highly dominated by not yet characterized bacteria and exhibits the highest concentrations of short chain fatty acids (SCFA) [28]. As broilers age, their caecal microbiota becomes more diverse. Out of 50 genera detected on day zero post-hatching the caecal genera increased to above 200 on day 42 post-hatching [29]. Temporal fluctuations occur particularly in the fecal microbiota due to the random emptying of the GIT section [30].

Previous studies of chicken broilers focused on lumen samples neglecting the mucosa that is mainly composed of mucins and glycans which promote colonization by distinct groups of microorganisms. Studies in humans, mice, rats, macaques, pigs and cows showed a divergence between lumen- and mucosa-associated microbiota structures [[38], [39], [40], [41]]. In contrast to the continuous flux of nutrients in the lumen, the mucosa is expected to show a more stable balance of nutrients which may represent a selective criterion for certain bacterial species [39]. A recent comparison between lumen and mucosa associated microorganisms revealed a much greater microbial community richness in the mucosa, particularly in the ileum and caecum of broiler chickens [13]. *Pseudomonas* spp. were detected in the ileal mucosa but not in the lumen. These species have the ability to hydrolyze phytate, degrade starch and in soils they are known to improve plant phosphorus availability [31]. Species belonging to the genera of *Clostridium* XI and *Ralstonia* were present in higher abundance in mucosa samples, while *Lactobacillus* sp. were three times more abundant in the ileal lumen. High abundance of commensal *Clostridium* XI species might induce a greater bacterial translocation from the ileal mucosa to the lymph nodes triggering an inflammatory immune response in the lymphatic tissues as previously described for pigs [32]. The caecum is the most diverse gut section and distinct community structures were observed in the lumen and mucosa samples. While the genera *Anaeroplasma*, *Oscillibacter*, *Papillibacter*, *Peptococcus* and *Subdoligranulum* were more abundant in the lumen, *Lactobacillus*, *Ruminococcus*, *Turicibacter*, *Clostridium* XlVa and *Clostridium* XlVb were detected in higher abundances in the mucosa. These observations emphasized the importance of studying the variations between the bacterial communities of the lumen and mucosa throughout the different sections of the GIT to improve our understanding of host-microbe interactions.

The majority of studies based on targeted 16S rRNA gene sequencing demonstrated effects of specific diet supplementations on the microbiota: probiotics, prebiotics and synbiotics [14,33,34]; Ca, P, phytases [13,28,35] and sodium butyrate [17]. Other studies characterized the different sections of the GIT of broilers under varying conditions analyzing bird performance [[36], [37], [38]], antimicrobial feed additives [11,39,40], gender [41], disease [42], host genetics [18,41], spatial microbial diversity [30,43] and meat flavor [33]. However, this is only a sparse depiction of the complexity and variability that exists within the highly diverse feeding and management conditions in animal production. Moreover, these investigations could not access the functional profiles and the activity of the respective microbiotas.

### 2.2 Metagenomic Shotgun Sequencing

Metagenomics, as a procedure to describe the collection of genomes and corresponding genes of a given ecosystem, permits the characterization of the potential bacterial functionality in specific environments [44]. Only a few metagenomics studies made the effort to answer the question: What are microorganisms actually doing in the chickens' GIT? (Table 1). The respective studies employed Roche 454-pyrosequencing and Illumina MiSeq or HiSeq platforms [11,45] to obtain the respective sequence information. It is expected that in the future more studies will rely on the Illumina technology since it grants a more convenient treatment of sequencing errors through computational approaches [19] including as well a greater coverage and yield which decrease systematic errors and costs [46]. Bioinformatic analyses include sequence assembly using the Velvet assembly tool (CLC workbench, Newbler version 3.0, BaseSpace) or automatic annotation by MG-RAST. The basic local alignment search tool (BLAST) is used to define functional groups and bacterial taxa. Subsequently, gene functions may be analyzed using the Kyoto Encyclopedia of Genes and Genomes (KEGG) or cluster of orthologous genes (COG). Up to now, metagenomics studies of the chickens' GIT have focused on the functions of the caecum [5], the response mechanisms to challenge by pathogens [45], the prominent role of the microbiota regarding performance parameters [47], comparisons between fat and lean lines [15], depiction of the virulome [45,48,49] and of antibiotic resistance genes [50,51] (Table 1). To obtain information about the taxonomic distribution of the microbial communities, studies focused on the most prevalent phylotypes representing the functional gene composition of the metagenome. The most abundant caecal phylotypes belong to the phyla of Firmicutes (44–55%) and Bacteroidetes (22–42%) [45], followed by the low abundant phyla of Actinobacteria, Chlorobi, Deferribacteres, Fusobacteria, Proteobacteria and Verrucomicrobia [45]. Analysis of environmental gene tags (EGTs) revealed that approximately 1% of the sequences belong to Archaea, mostly to Euryarchaeota but as well as to Eukaryota, Fungi and Viridiplantae [45]. The caecal metagenome of chickens challenged by *Campylobacter jejuni* revealed that mobile elements were a contributing factor to the functional components of the microbiota and that these genes were associated with virulence clustering according to the environment [45].

**Table 1:** Summary of the studies investigating chicken microbiome in respect to the influence of feeding impact with metagenomics and metaproteomics methodologies.

Metagenome details	Study focus	Diet	GIT sections	Number of samples	Sampling time	Reference
GS-FLX sequencing Reads: 1.291.219 Av. length: 234–399 bp	Effects of subtherapeutic levels of virginia and tylosin and the coccidial monensin on bacteria composition from the chicken caecum (metagenomics and 16S)	7 d of basal diet followed by supplementation with: Monensin sodium, Monensin sodium + virginiamycin or tylosin phosphate	Caeca	Pooled samples per treatment	0 d, 7 d, 14 d and 35 d Ross × Ross chickens	[11]
Illumina MiSeq2000 Reads: 81.772.788 Av. length: 110 bp	Elucidation of the functions of the cecal microbiota and characterization of the community profile (metagenomics and 16S)	Wheat based diet with 5% maize	Caeca	20	42 d of Ross broilers	[5]
Illumina HiSeq2000 Reads: 52.485.882 Av. length: 100 bp	Study if variation of fatness is link to the composition of gut microbial metagenome. Lean and fat lines were employed.	Commercial diet	Feces	29	Fat and lean lines. Weeks 37 to 40	[54]
Illumina HiSeq2000 Reads: 37.9 million (per sample) Av. length: 100 bp	Comparison of two lines of chickens (fat and lean). Understand the influence of the host in the gut microbiota	Commercial diet	Feces	6	35 wks	[15]
Illumina HiSeq 2000 Av. length: 100 bp	Antibiotic resistance genes annotation from metagenome of pig, chicken and human and its co-occurrence with associated genetic elements	Commercial diet	Feces	8	20 d and 80 d	[51]
454 sequencing Reads: 24–30 million Av. length: 100 bp	Phylotype and functional gene content characterization before and after inoculation with *Campylobacter jejuni*	Commercial diet and 14 days post-hatching one group was challenged with 10^5 CFU of *C. jejuni*	Caeca	2	28 d (14 d of challenge)	[45]
GS-FLX sequencing Reads: 94.926 (low FCR); 63.891 (high FCR) Av. length: 227 bp	Characterization of poultry fecal microbiome of low and high feed conversion ratio (FCR) broilers	Commercial diet	Feces	Pooled samples for high and low FCR	49 d broiler strain MY	[53]
Illumina HiSeq 2000 Reads: 4.737.146	Investigate the occurrence, diversity and abundance of antibiotic resistance genes in feces of layers and broilers	Commercial diet	Feces	Pooled samples	6 wk broilers and 52 wk laying hens	[50]
Metaproteomics	Microbial composition in the healthy chicken gut	Attlee's nonmedicated poultry feed	Feces	Pooled samples	18 wk white leghorn chickens	[57]
	Dietary effect of mineral phosphorous and microbial phytase	3 diets with P from plant sources (BD−), 3 diets with P supplementation (BD+). BD− and BD+ supplemented with 0, 500 and 12,500 U/kg of *E. coli* phytase	Crop Caeca	Pooled samples per treatment	25 d broilers Ross 308	[58]

The caecum consists of two long anoxic blind sacs that harbor a microbiota dominated by carbohydrate metabolism with lower occurrence of respirational genes [45]. Fermentation pathways in this GIT section lead to the production of short chain fatty acids (SCFA), which are further absorbed and assimilated by the host [52]. Sergeant et al. [5] identified butyrate-producing genes for enzymes like 3-hydroxybutyril-CoA dehydrogenase, phosphate butyryl transferase and butyrate kinase. Moreover, acetate-CoA transferase responsible for acetate synthesis and gene clusters that encode for the beta, gamma and delta subunits of methylmalonil-CoA decarboxylase, which is involved in the formation of propionate, were found to be present [5]. Twelve putative uptake hydrogenases produced by *Megamonas*, *Helicobacter* and *Campylobacter* were also identified in the caeca. The authors speculated that the respective hydrogenases have the potential to serve as hydrogen sinks that facilitate succinate production [5]. High proportions of the metagenomic sequences encoded for glycosyl hydrolase domains of glucanases, which act on oligosaccharides and are produced by bacteria belonging to Negativicutes and Lentisphaera, and further of endoglucanases that degrade polymers like cellulose and xylan, synthesized by Actinobacteria, Clostridia and Bacteroidia [5]. Furthermore, genes involved in cell wall metabolism and virulence were found to be present [45]. Regarding supplementation with antibiotics, it was reported that diets containing monensin and antibiotic growth promoters have no influence on the broadest functional classification of the microbes present in the caeca when compared to control diets. However, a combination of monensin with virginiamycin and tylosin increased the presence of conjugative secretion systems, specifically for plasmid types commonly found in *E. coli*. However, antibiotic resistance genes were also present in control and treatment groups [11]. As experiments are usually carried out in standardized and controlled animal facilities, conclusions about antibiotic resistance should be carefully stated. A comparison of metagenomes from feces of chickens, pigs and humans showed a high homology to tetracycline genes (*tet*A) and the presence of gene combinations of individual resistance elements, which encode for resistance to beta-lactams, aminoglycosides, macrolides and multidrug [51]. These findings demonstrated that there is a potential risk in the dissemination of the antibiotic resistance between farming animals and humans, therefore these supplementations should be considered cautiously.

Metagenomic analyses of fecal samples found Proteobacteria to be the most abundant phylum (47–79%) followed by Firmicutes (12–28%) and Bacteroidetes (7–27%) [50,53]. Animals with a high feed conversion ratio (FCR) exhibited a higher abundance of the genera of *Acinetobacter*, *Bacteroides*, *Streptococcus*, *Clostridium* and *Lactobacillus* whereas in low FCR animals *Escherichia*, *Shigella* and *Salmonella* were more abundant [53]. Regarding lean lines, the same study revealed an enrichment of microbial functions in four classes of the category transport and metabolism of the clusters of orthologous groups: amino acid, nucleotide, coenzyme and lipids [54]. Another study supported that lean lines exhibit an increase in lipid storage, including the peroxisome activated receptor (PPAR) and the citrate cycle, which unifies the carbohydrate, lipid and protein metabolism [15]. The same functions were detected in human studies that related the microbiome to the development and progression of obesity, besides the citrate synthase activity [15,55,56]. The limited amount of studies and samples that have been analyzed so far reveals that metagenomic approaches are still not affordable for a great percentage of groups studying the chickens' GIT. However, additional research is necessary, as microbial communities have an impact on the chickens' metabolism, immune homeostasis and colonization resistance.

### 2.3 Metaproteomics

Advances in DNA and RNA sequencing caused a boost in the discipline of metaproteomics. The increased availability of sequenced genomes and metagenomes promotes the identification and characterization of an increased number of proteins that are expressed by specific microorganisms in a given sample. Metaproteomic studies of the chickens gut are scarcely available in the literature. Up to now, only two studies applied this technique to characterize the adaptation of the chickens' gastrointestinal microbiota to a specific challenge [57,58] (Table 1).

Another study by Polansky et al. investigated the chickens' caecal microbiome following inoculation with caecal extracts from chickens of different ages, in order to elucidate the colonization patterns and predict the most promising probiotic genera for caecal colonization of newly hatched chickens [59].

Tang et al. studied two fecal samples of 18-week-old white leghorn chickens [57] identifying 3673 proteins of 799 different genera. The most abundant bacterial genus was *Lactobacillus* (11% of total proteins) followed by *Clostridium* (4% of total proteins) and *Streptococcus* (2% of total proteins). The findings could not be correlated with the 16S rRNA gene sequencing analysis that exhibited higher abundances of Clostridiales (25% of total sequences), Bacteroidaceae (21% of total sequences) and Lactobacillaceae (19% of total sequences). GroEL, a stress-related protein, was the most abundant protein followed by glyceraldehyde-3-phosphate dehydrogenase which is a key enzyme in glycolysis and gluconeogenesis [57].

**Fig. 1:**
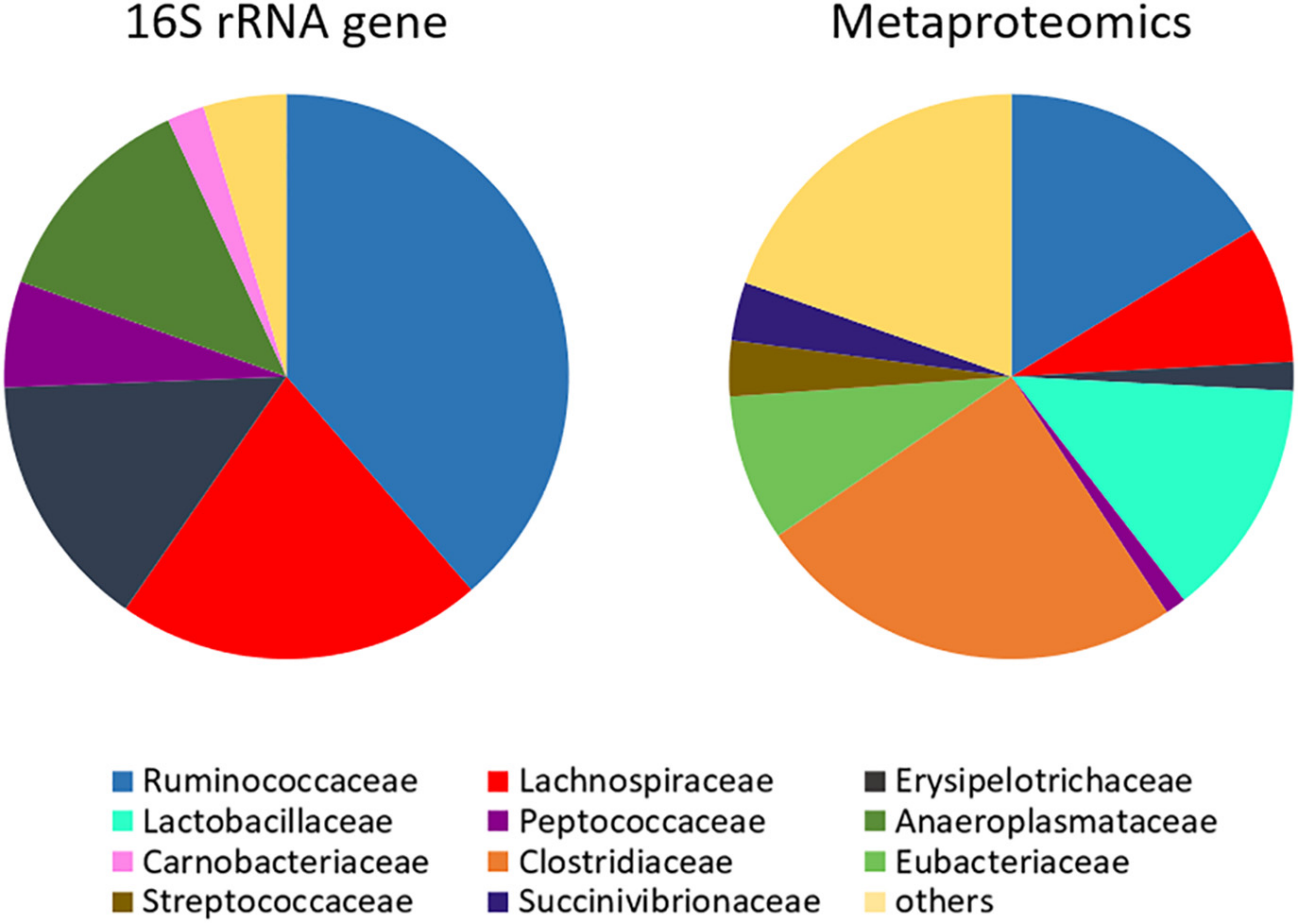
Families with more than 1% of abundance obtained from caeca content with 16S rRNA gene [13], and metaproteomic [58] analyses.

The second study by Tilocca et al. investigated the influence of supplementing inorganic phosphorous (P) and/or microbial phytases on the formation of inositol phosphates and the intestinal microbiome [58]. Crop and caeca contents of 48 animals were sampled and pooled per pen and dietary treatment resulting in 24 analyzed samples. A total of 381 bacterial proteins were identified in the crop with most identified proteins being assigned to the Lactobacillaceae family, disregarding the dietary treatments. In diets supplemented with P, the number of proteins belonging to the Veillonellaceae family increased [58]. In the caeca, a total of 1719 proteins were identified. Proteins synthesized by species of the Eubacteriaceae family appeared in lower abundance in diets supplemented with P while proteins of the Bacteroidaceae family increased in abundance. The number of proteins of the Ruminococcaceae family was higher in diets with microbial phytase supplementation. A lack of P and microbial phytase supplementation caused a stressed microbial community with exclusive occurrence of COG categories at low relative abundances, while P and microbial phytase supplementation showed a prosperous microbiota assemblage. The authors identified a low number of host proteins in the crop (248) and in the caeca (405), emphasizing that an accurate sample preparation is essential to enrich proteins of prokaryotic microorganisms to improve the numbers of total proteins detected by mass spectrometry-based metaproteomics [58]. Fig. 1 shows a comparison of the bacterial families detected in caecal samples from identical basal diets by targeted amplicon sequencing [13] and metaproteomics [58]. There was a great discrepancy in the relative abundance of identified families. Ruminococcaceae, Lachnospiraceae, Erysipelotrichaceae, Peptococcaceae, Anaeroplasmataceae and Carnobacteriaceae were detected in higher abundance by targeted amplicon sequencing, while Lactobacillaceae, Clostridiaceae, Eubacteriaceae, Streptococcaceae and Succinovibrionaceae were found to be more abundant in the metaproteomic study. Methodological biases such as varying numbers of 16S rRNA gene copies and a higher sensitivity of the targeted amplicon sequencing approach in regard to low abundant species as well as a lack of genomic sequences in databases required for proteomic approaches [57,58] could be an explanation for these results.

The advantage of metaproteomics and also metatranscriptomics is to gain more precise insights into the actual functions carried out by microorganisms of a microbiome, especially when compared to the rather vague predictions based on 16S rRNA genes or metagenomics. In addition, the co-extraction of host RNA or proteins may as well be beneficial to gain concomitant information about the host status, although a high quantity of these host biomolecules can clearly impair the analysis of the microbiome. Thus, a balanced methodological workflow has to be established for proper application of the respective meta-omic approaches.

## 3 Chicken Feeding and Its Influence on the Microbiota

The nutrition of chickens is based on plant diets that are supplemented with a variety of amino acids, minerals, vitamins, enzymes, pre-, pro- and anti-biotics to improve growth performance. The respective supplements may replace nutrients or improve the accessibility of nutrients that are not easily assimilated by the animals due to the varying digestibility of substrates. The use of a high percentage of animal protein is avoided in chicken diets because it increases the abundance of *Clostridium perfringens* in the GIT which is a predisposing factor for necrotic enteritis in chickens [60]. The ban of antibiotics as growth promoters by the European Union and its potential restriction in other countries [61] intensified the search for alternatives to improve growth performance and to avoid a raise in animal diseases such as necrotic enteritis, gut dysbiosis, diarrhea, loss of appetite and dysregulation of the immune system [62].

Poultry diets have a tremendous impact on the gut microbiome in regard to diversity and composition. Varying dietary compositions influence growth performance in the intensive growing period. Cereal types comprise different concentrations of soluble non-starch polysaccharides (NSP) such as arabinoxylans which occur in higher concentrations in wheat as when compared to maize [63]. Diets with high levels of NSP, such as barley-, rye- and wheat-based diets, improve lumen viscosity, increase the retention time of feed and reduce nutrient digestibility [64]. Short retention time selects for rather fast growing bacteria which adhere to the epithelium [65]. Such conditions favor the colonization of *Clostridium perfringens* and prompt the occurrence of necrotic enteritis disease [65]. The inclusion of feed additives in the diet helps the modulation of gut microbiome by stimulating the growth of specific microorganisms that improve gut health. Particularly, the enzymes xylanase and β-glucanase are known to foment the growth of lactic acid bacteria. Those bacteria have the ability to adhere to the gut epithelium and compete with pathogens for its colonization while decreasing lumen viscosity [65,66].

High amounts of phytic acid in plant based diets and derived feedstuffs and the limited presence of endogenous phytase in the GIT mucosa of chickens leads to the supplementation with microbial phytases that are highly beneficial since catalyzing the hydrolysis of phosphate groups from the inositol ring [67]. In substrates like rapeseed cake, phytase supplementation improves the apparent total protein digestibility [68]. During the last years several studies have been designed to address the influence of phytase supplementation on the availability and interaction with P and Ca in regard to the microbial communities and to meet the animal requirements. Diets supplemented with microbial phytases increase the release of P and Ca from phytate and hence reduce the supplementation of inorganic phosphate and Ca required in poultry diets [35]. In the crop, phytase promotes the abundance of Aeromonadaceae and Flavobacteriaceae while reducing the dominance of *Lactobacillus* [69]. Furthermore, DAPI counts of bacteria revealed that the presence of phytase in the diet, with adequate or deficient levels of Ca and P, enhances the total number of bacteria [35]. Phytase supplementation increases the abundance of *Lactobacillus* sp., *Clostridium leptum* and *Enterococcus* sp. in the ileum [35]. Monocalcium phosphate, an inorganic compound generally added to diets, increases the presence of members of the Clostridiales order and the Bacteroidaceae family [69].

Organic acids, such as acetic acid, propionic acid and butyric acid [70], were used to selectively stimulate the permanence of beneficial bacterial species and various studies reported fluctuations regarding gain of weight, feed intake and feed conversion ratio [[71], [72], [73], [74]]. Sodium butyrate is a common dietary supplement and is transformed to butyric acid by the chicken's metabolism. It affects the development of the gut epithelium and promotes the presence of symbiotic bacteria. A decreasing pH in crop and gizzard favors the establishment of lactic acid producing bacteria including *Lactobacillus* spp. and *Bifidobacterium* spp. [75,76], while reducing the colonization by harmful bacteria like *Salmonella enterica* and *Campylobacter jejuni* [77].

Prebiotics are non-digestible oligosaccharides that show a positive effect on the host by stimulating the growth of certain bacteria. They serve as a source of nutrients for commensal microbes and can mislead pathogenic bacteria to attach to the oligosaccharide and to be excreted before attaching to the mucosa and causing infections [78]. Xylo-oligosaccharides are products of the hydrolytic degradation of arabinoxylans and have been used in broiler diets as prebiotics. Their main functions are associated with the increment of villus length in the ileum and the promotion of beneficial microbial groups in the GIT. In the colon, xylo-oligosaccharides increase the presence of *Lactobacillus* and in the caeca the *Clostridium* cluster XIVa which is known to possess genes related to butyrate production such as the butyryl coenzyme A and acetate CoA transferase [79]. Another source of oligosaccharides includes the ones derived from palm kernel expeller. It is assumed that improves the immune responses due to the increase of IgA and IgM along with the promotion of *Bifidobacterium* and a reduction of *Salmonella* [80]. Alternatively, lactulose, a synthetic disaccharide prebiotic, can stimulate the growth of *Lactobacillus* and *Bifidobacterium* and reduce pro-carcinogenic activity based on enzymes such as azoreductase or 7-alpha-dehydroxylase [81]. Prebiotics produced from yeast cells and cell walls are used due to the positive effect on gut health and microbiota modulation. Beta-D-glucan and mannan-oligosaccharides, components of this supplement, bind to the receptor mannose-specific type-1 fimbriae and prevent pathogen colonization while favoring the genus *Faecalibacterium* which is commonly associated with gut health [82].

Probiotics are living microorganisms that improve gut health and animal performance if added to the diets in adequate amounts. These microorganisms compete with pathogenic bacteria for adhesion sites at the intestinal epithelium [83]. Moreover, mechanisms of action from probiotics consist of the enhancement of activity of digestive enzymes like proteases, lipases and amylases [84], the improvement of mucosa ultrastructure, thus also increasing nutrient absorption [85]. The use of the probiotic *Lactobacillus plantarum* P-8 in broiler diets enhances the immune response, weight gain, feed efficiency and feed intake. Moreover, metabolic activity and nutrient utilization are improved and furthermore, the fecal microbial composition is modulated [62]. *Enterococcus faecium* supplementation (0.5% of the total diet) reduces the microbial counts of *Salmonella* and increases body weight gain and breast muscle yield [85]. *Bacillus* sp. can be delivered in pelleted feeds due to their stability and heat resistance which improves the production of enzymes like proteases, amylases and lipases positively influencing growth performance. In addition, *Bacillus* sp., also impact the small intestinal micro structure with an increase of villous height and *Lactobacillus* and *Bifidobacterium* counts in the caeca. Its supplementation decreases the presence of harmful bacteria such as *E. coli* and *Salmonella* sp. [86].

Synbiotics combine the effects of pre- and probiotics. Such mixtures improve the implantation and survival of the supplemented bacteria in the GIT [87]. Synbiotics showed a great efficacy in the reduction of *C. jejuni*, which causes zoonosis frequently and provokes a strong inflammatory response [88]. The combination of *Bifidobacterium longum* PCB133 with a xylo-oligosaccharide (XOS) successfully reduced the load of *Campylobacter* spp. and *C. jejuni* [89]. It has been demonstrated that the delivery of synbiotics by *in ovo* technology [90] can modulate gene expression levels in immune related tissues and gut structures. The inoculation of galactooligosaccharides and *L. salivarius* or raffinose and *L. plantarum* increased the absorbent surface of duodenum and jejunum [91,92].

Metabolites synthesized from probiotics are referred to as “postbiotics” and represent an alternative since exerting the positive effect of probiotics without applying living cells [93]. As an example, *Lactobacillus* sp., are able to produce organic acids and bacteriocins that promote the presence of lactic acid bacteria. Consequently, there is a decrease of pH and counts of enterobacteria, an intensification of mRNA IGF1 expression which is an indicator for body composition, growth, fat deposition and metabolic activities, and mRNA GHR gene which plays a role as mediator of body size [93].

Innovative dietary supplements, announced as an environmental friendly solution, appear in the market with a lower cost. Earthworm meal can positively affect the growth performance of chickens, and increases the concentrations of Ca and P in the blood [94]. Another dietary intervention includes the addition of dry whey powder, a co-product of cheese industry, acting as a prebiotic for gut microflora due to its high content of lactose and protein quality, and exhibiting a positive influence on the bird performance from early to later growth stages [14]. Essential oils of oregano and laurel are being explored due to their antioxidant and antimicrobial characteristics and the enhanced digestibility based on the stimulation of endogenous enzymes, nitrogen absorption and inhibition of odor and ammonia [95]. These compounds were also shown to increase the body weight and FCR and exhibiting less mortality when compared to the control group. In ileum and caecum, they modulate the microbiota towards an increase of *Lactobacillus* and *Bifidobacteria* counts. Essential oils of oregano and laurel enhance villus height, antioxidant capacity of breast and thigh meat [95]. Moreover, a resin from the plant *Boswellia* *serrata* was approved as a safe additive in poultry production and exhibited therapeutic capabilities including anti-inflammatory and antibacterial effects which stabilize the intestinal functions. A better digestive efficiency was achieved considering dry and organic matter and an increase of the genus of *Lactobacillus* and *Enterococcus* [96] was observed.

## 4 Future Perspectives

The current state of knowledge about the chickens' intestinal microbiota is mainly based on the general inventory of the bacterial populations. Variations of the community structures were mainly investigated with respect to different feeding strategies and the influence of pathogenic species, but the question arises if the results obtained by numerous studies are comparable to each other. Although experiments are commonly standardized and based on identical breeds such as Ross 308 broilers, there is a lot of deviation concerning the subsequent processing like DNA extraction and selection of the variable region for amplification. Different laboratory protocols lead to incomparable results. Thus, a standardized protocol as it is available in human microbiota research should be established in chicken microbiome research to obtain comparable datasets. Another issue regarding the experimental design is the pooling of samples from different animals which concerns numerous studies. Borda-Molina et al. [13] reported a high individuality of the microbiota structure of single birds despite the fact that the animals originated from the same breeder and were housed under the same conditions. Consequently, pooling of samples can mimic changes in the microbiota composition which otherwise would not be visible. Regarding the sampling procedure itself, the study mentioned above also emphasized the importance of sampling mucosa and lumen digesta separately to obtain a more complete representation of the microbiota. A combination with a predictive functionality may depict the microbial processes that are running at the host interface and identify microorganisms which are most relevant to the host animal. This may represent a starting point to further study the interaction between microbiota and host.

**Fig. 2:**
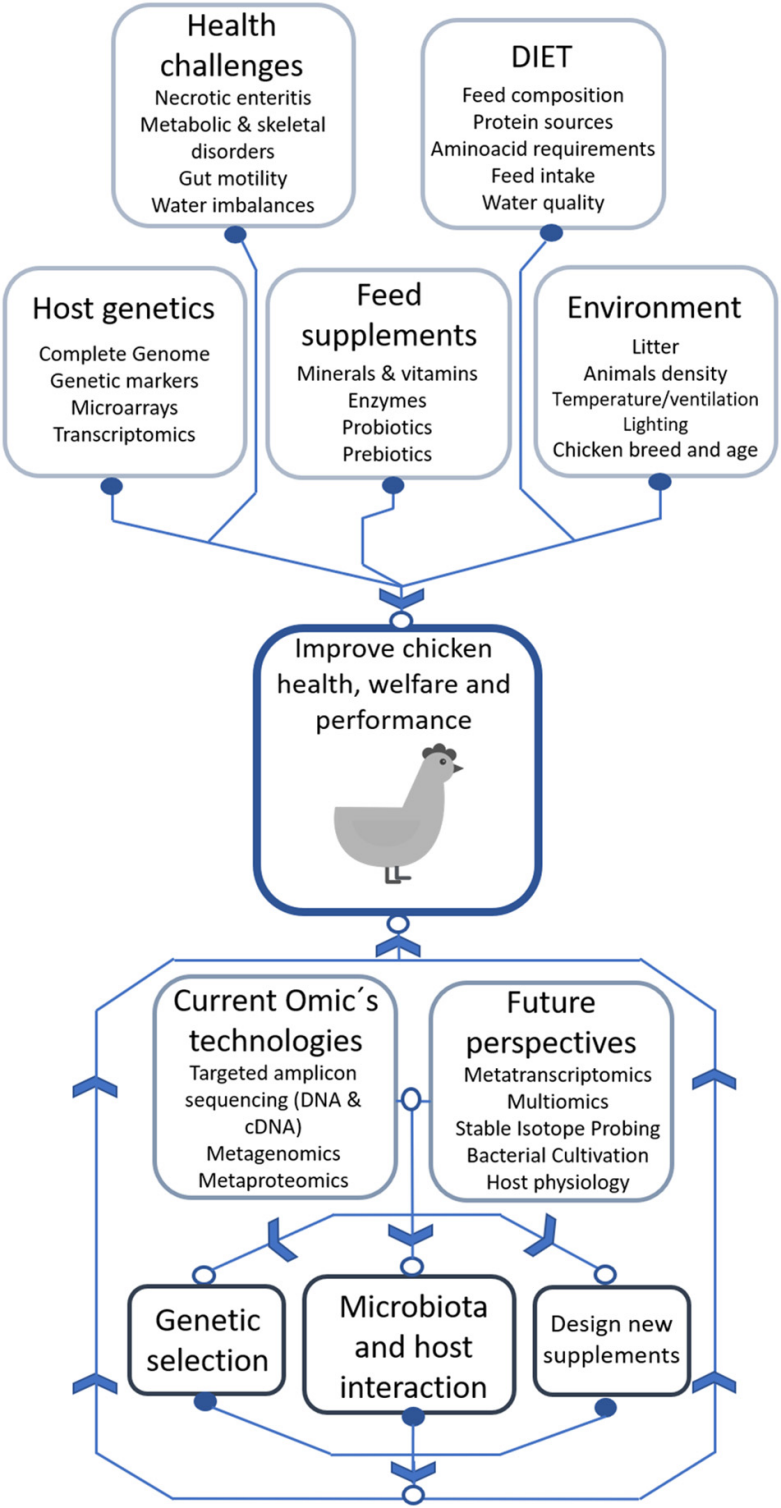
Overview of the factors affecting chicken health, welfare and performance and future perspectives in the analysis of the chicken microbiome.

So far studies of the chicken microbiota are mainly performed using 16S rRNA gene amplicon sequencing and metaproteomics. The use of metatranscriptomics and metabolomics, and the combination of all are still at the very beginning but have the potential to move from predictive analyses to more accurate descriptions of the actual microbial activities. Another important issue is the limited culture collection of strains inhabiting the GIT of chickens. An increase in bacterial cultures and their genetic and biochemical characterization would strongly support the Omics data evaluation. To reach this, cultivation strategies should be created which consider the demand of co-culturing or host-derived substrates as it was done for the mouse and humans [97] (Fig. 2).

So far, the main focus of microbiota research in chickens has been on understanding how the microbiota is changing under defined feeding strategies and how this influences the performance of the broilers and laying hens. Another focus is the control of pathogens under production conditions. For both interests and many others, gnotobiotic chickens could be of great importance. They are available and already used to study the expression of host enzymes [98]. Although the handling of gnotobiotic chickens is also challenging, including facts like faster growth, higher caloric intake, abnormal gut motility, thinner intestinal wall or high urea/uric acid ratio in feces and metabolism and recycling of bile acids [[99], [100], [101], [102]], they should be used for infection and feed digestion studies with defined microbial cultures structures to gain more insights into the function of the microbiome and the interaction with the host in the future.
